# Adipose tissue-derived stromal vascular fraction in regenerative medicine: a brief review on biology and translation

**DOI:** 10.1186/s13287-017-0598-y

**Published:** 2017-06-15

**Authors:** Pablo Bora, Anish S. Majumdar

**Affiliations:** 1 Stempeutics Research Private Limited, Akshay Tech Park, # 72&73, 2nd Floor, EPIP Zone, Phase 1, Whitefield, Bangalore, 560066 India; 2 Present Address: Department of Molecular Biology & Genetics, Faculty of Science, Jihočeská univerzita v Českých Budějovicích (University of South Bohemia), Branišovská 31, 37005 České Budějovice, Czech Republic

**Keywords:** Regenerative medicine, Multipotent-stromal cells, Stromal vascular fraction, Point-of-care biomedical devices, CD34, Regulation of stem cell therapeutics

## Abstract

Adipose/fat tissue provides an abundant source of stromal vascular fraction (SVF) cells for immediate administration and can also give rise to a substantial number of cultured, multipotent adipose-derived stromal cells (ADSCs). Recently, both SVF and ADSCs have gained wide-ranging translational significance in regenerative medicine. Initially used for cosmetic breast enhancement, this mode of treatment has found use in many diseases involving immune disorders, tissue degeneration, and ischaemic conditions. In this review, we try to address several important aspects of this field, outlining the biology, technology, translation, and challenges related to SVF- and ADSC-based therapies. Starting from the basics of SVF and ADSC isolation, we touch upon recently developed technologies, addressing elements of novel methods and devices under development for point-of-care isolation of SVF. Characterisation of SVF cells and ADSCs is also an evolving area and we look into unusual expression of CD34 antigen as an interesting marker for such purposes. Based on reports involving different cells of the SVF, we draw a potential mode of action, focussing on angiogenesis since it involves multiple cells, unlike immunomodulation which is governed predominantly by ADSCs. We have looked into the latest research, experimental therapies, and clinical trials which are utilising SVF/ADSCs in conditions such as multiple sclerosis, Crohn’s disease, peripheral neuropathy, osteoarthritis, diabetic foot ulcer, and so forth. However, problems have arisen with regards to the lack of proper regulatory guidelines for such therapies and, since the introduction of US Food and Drug Administration draft guidelines and the Reliable and Effective Growth for Regenerative Health Options that Improve Wellness (REGROW) Act, the debate became more public with regards to safe and efficacious use of these cells.

## Background

Adipose-derived stem/stromal cells (ADSCs) were first characterised in 2001, and have since been widely studied and used as a major source of cells with regenerative potential, with characteristics similar to that of mesenchymal stem/stromal cells (MSCs) [1–4]. ADSCs are isolated as part of the aqueous fraction derived from enzymatic digestion of lipoaspirate (the product of liposuction). This aqueous fraction, a combination of ADSCs, endothelial precursor cells (EPCs), endothelial cells (ECs), macrophages, smooth muscle cells, lymphocytes, pericytes, and pre-adipocytes among others, is what is known as the stromal vascular fraction (SVF).

ADSCs, like MSCs, have shown promise in regenerative and reconstructive medicine [5–8]. Recent advances in the area of tissue regeneration have put SVF on a par and at times even above ADSCs [9–17]. For instance, in a study of erectile function in a rat model of cavernous nerve injury, SVF treatment showed superior statistically significant results compared to ADSC treatment alone, especially in smooth muscle/collagen ratio and in endothelial cell content [12]. The advantage of SVF over ADSCs is believed to be in two fundamental areas. Firstly, although similar in properties such as immunomodulation, anti-inflammatory, angiogenesis, and so forth, the distinctive, heterogeneous cellular composition of SVF may be responsible for the better therapeutic outcome observed in comparative animal studies [9–12]. Secondly, unlike ADSCs, SVF is much more easily acquired, without the need for any cell separation or culturing conditions. Thus, the therapeutic cellular product is instantaneously obtained and has minimal contact with reagents making it comparatively safer and subject to the fulfilment of lesser regulatory criteria. It should be noted that, whereas ADSCs find utility in both allogeneic and autologous treatments, SVF, owing to the presence of various cell types known to cause immunological rejection, is suitable for autologous treatments only.

Although almost all ADSCs are derived from the white adipose tissue (WAT), as covered in this review, the identification of progenitor cells in brown adipose tissue (BAT) of adult humans is fascinating and worth a mention [18, 19]. Termed as BADSCs (brown adipose-derived stem cells), these have been isolated from BAT deposits present in relatively inaccessible regions such as the mediastinum, and are capable of differentiating to metabolically active BA cells with differences in surface antigen expression as compared to WAT-originating ADSCs [18]. Current understanding of WAT and BAT define these cells with distinct functionalities, and thus translational avenues for ADSCs from either source should be compared to identify specific therapeutic targets and potential advantage of one over the other. Understanding of the molecular mechanisms behind either cell fate and the possibility of inter-conversion are interesting avenues of research with basic and translational implications [20, 21].

Despite the potential of SVF in regenerative medicine there are challenges to overcome. First is isolation of SVF, which needs a specialised infrastructure such as a clean room facility, equipment, reagents, and technical capabilities. These conditions limit the reach of SVF to only major hospitals in tier 1/2 cities, especially in a country such as India. In this regard, the up and coming point-of-care biomedical devices which can take lipoaspirate as their input and produce sterile, injectable SVF as output will be beneficial. Secondly, the method of isolating SVF is a vital roadblock in the approved use of SVF for therapeutic applications. Digestion of lipoaspirate is achieved by collagenase, and the presence of collagenase in the injectable product does not bode well with regulatory authorities such as the US Food and Drug Administration (FDA) [3]. Consequently, alternative methods are being explored with some encouraging outcomes [22–25]. Finally, characterisation of the regenerative cells of SVF has not reached a wide consensus. Organisations such as the International Federation for Adipose Therapeutics and Science (IFATS) and the International Society of Cellular Therapy (ISCT) have been updating the surface antigen-based definition of SVF cells, where CD34 antigen, primarily associated with haematopoietic stem cells (HSCs), became an important marker of regenerative, MSC-like cells of the SVF [1, 26, 27].

In this review, using the broader topics of isolation and characterisation of SVF, we will touch upon some of the challenges and innovations in the field and comment upon the future of SVF.

## Isolation of SVF

### Enzymatic isolation of SVF

The most widely used technique for the isolation of SVF from lipoaspirate is by digestion of the fatty portion of the lipoaspirate with collagenase, separating the contents into two distinct phases: the floating mature adipocytes fraction, and the cellular components of interest in the lower aqueous fraction [17, 28]. This separation can be enhanced by centrifugation; nevertheless, comparable separation can be achieved by gravity-based phase separation and filtration [29]. Although centrifugation is more efficient, it will also pellet down all the cells present, while filtration can be designed to capture only the important cell types based on size, thus enriching the specific cellular cocktail.

Centrifugation of the aqueous fraction yields a reddish pellet which contains SVF cells. Erythrocytes, a major contaminant present in the SVF pellet, can be lysed to isolate a purer population of ADSCs and/or SVF cells if intended for in vitro expansion [7, 30].

### Non-enzymatic isolation of SVF

In view of the regulatory questions relating to enzymatic isolation, it is important to look into alternative methods for isolating SVF and compare these with the conventional methods [3, 24, 25]. Most of these techniques involve mechanical agitation which breaks down the adipose tissue and releases the stromal cells. As expected, the cellular yield from mechanical procedures are much lower compared to enzymatic methods, as cells of the adipose tissue tightly bound by collagen will not be easily released by mechanical action alone [24].

A novel method of mechanical agitation was recently defined by Tonnard et al. [23]. The injectable product, termed as “nanofat”, was obtained by emulsification and filtration of the lipoaspirate. Although termed as nanofat grafting, in effect no viable adipose cells survived the emulsification process, but the graft was rich in CD34^+^ ADSCs. The efficacy and properties of nanofat have been demonstrated in multiple case studies related to skin rejuvenation, scar healing, skin grafting for wound management, and treating vulvar lichen sclerosus (VLS), a chronic inflammatory disease of the anogenital area, and also by standard ADSC-related phenotypic and differentiation studies [23, 31, 32]. Owing to the simplicity of the technique, it might be amenable to scaling up by simply using the desired volume of syringe and/or using multiple syringes as required.

The effect of the emulsification process on other cells of interest, normally found in enzymatically processed SVF, remains to be seen. Combining such techniques with centrifugation or filtration can yield products highly concentrated with ADSCs, thus eliminating enzymatic digestion, reducing process time, cost, and respective regulatory constraints.

### Automated devices for point-of-care isolation of SVF

The infrastructure, expertise, and consumables required for the conventional method of SVF isolation is not commonplace in most health-care facilities. Cosmetic surgery, being at the upper-end of medical expenditure, is the largest consumer of SVF and related products, but the actual scope is much wider [3]. Thus, it is unfortunate that the benefits of this very simple technology have not reached full potential. This gap can be overcome by automated, point-of-care biomedical devices, which can produce injectable SVF from lipoaspirate.

Such developments have been underway for quite some time, although mostly still in trial stages, with Cytori’s (San Diego, USA) Celution® being the first system [33]. Currently, about 30 different automated and semi-automated systems are under development [22]. The technologies and methodologies used vary, with most opting for the tried and tested enzymatic process. Stempeutics (Bangalore, India) has developed one such system, Stempeutron™, the proof-of-concept of which was reported in SundarRaj et al. [29]. Stempeutron™ uses the more efficient and conventional enzymatic digestion method and gravity-enabled separation of fatty and aqueous fraction followed by filtration of the aqueous fraction to achieve SVF isolation and concentration.

Since Stempeutron™ uses filtration we wanted to know the physical dimensions of SVF cells. As such, a list of cell sizes was not found while searching through the literature for this review and we resorted to mining for individual reports of cell size, surface area, and volume measurements. Table 1 summarises available cell diameter information accumulated from various reports [34–45]. The filtration system in Stempeutron™ is capable of capturing the majority of the therapeutically important cell types (Table 1) [3, 29]. Future developments might enable size-based enrichment of specific cellular populations, targeted towards specific diseases.

**Table 1:** Important components of SVF, respective sizes, and surface markers

Cell types of the SVF	Cell size range [in µm]*	Molecular markers^#^ [1, 26, 29]	
		Positive	Negative
ADSC	~10–25 μm and reported up to 200+ μm in culture [34–36]	CD34, CD73, CD13, CD90, CD105, CD29	CD31, CD45, CD144
EPC	~7–8 μm (smallest defined) [37]	CD34, CD31, CD133, CD146	CD45
EC	~10–30 μm [38]	CD31, FVIII	CD34
T regulatory cells	~7–12 μm [39, 40]	CD4, CD25, Foxp3, CD8	–
Macrophages	~20 μm [41]	CD45, CD14, CD34, CD206	–
Smooth muscle cells	~3–20 μm in width and 20–500 μm in length [42, 43]	Smooth muscle actin (SMA)	–
Pericytes	Up to ~70 μm in length [44]	CD146, CD90, CD73, CD44, CD29, CD13	CD34, CD45, CD56
Pre-adipocytes	~10 μm [45]	CD34	CD45, CD31, CD146

*Diameter; unless mentioned otherwise

The Table captures the approximate range of cell sizes as reported in different studies [34–45] and provides an overview of surface antigens for the respective cell type [1, 26, 29]. ^#^ Includes surface or CD markers, cytoplasmic and nuclear factors

*ADSC* adipose-derived stem/stromal cells, *EC* endothelial cells, *EPC* endothelial precursor cells, *SVF* stromal vascular fraction

## Characterisation of SVF

**Fig. 1:**
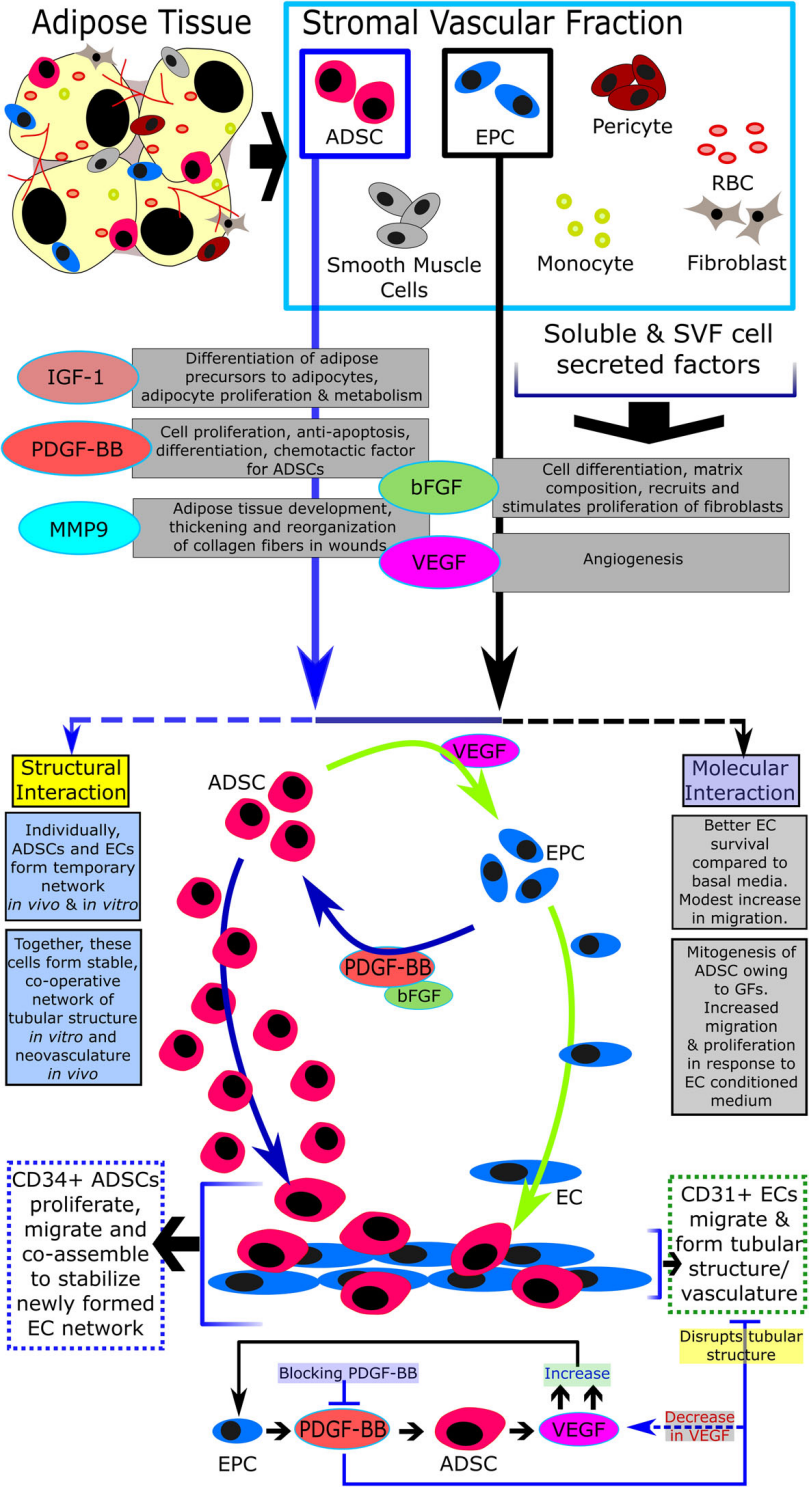
Potential mechanism of action of ADSCs and ECs present in SVF towards angiogenesis. Breakdown of adipose tissue releases many cell types, which together are termed SVF. The cells of the SVF can produce several bioactive soluble factors. ADSCs and EPCs, two important components of SVF, cross-talk via VEGF and PDGF-BB, respectively (among other components), to enable cell proliferation, homing towards injury, neovascularisation and other inter-connected outcomes. *ADSC* adipose-derived stromal cell, *bFGF* basic fibroblast growth factor, *EC* endothelial cell, *EPC* endothelial progenitor cell, *GF* growth factor, *IGF*-*1* insulin-like growth factor-1, *MMP* matrix metalloproteinase, *PDGF* platelet-derived growth factor, *RBC* red blood cell, *SVF* stromal vascular fraction, *VEGF* vascular endothelial growth factor

Criteria for characterising the cellular contents of SVF using surface antigen (cluster of differentiation (CD)) combinations is an evolving area of research as, within certain generally accepted norms, it differs between laboratories. A list of commonly used positive and negative markers identifying different cellular populations of SVF is provided in Table 1 [1, 26, 29]. Considering the variables present in isolation of SVF, such as the age of the patient, downstream processing, and so forth, the diversity observed between samples is quite understandable. However, if there is a relationship between the different ratios of cellular components present in SVF with its efficacy towards specific ailments, one might be able to come up with an optimum composition corresponding to the highest therapeutic efficacy. Traktuev et al. demonstrated that certain factors produced by ADSCs such as vascular endothelial growth factor (VEGF) help in migration, and that better survival of EPCs and correspondingly platelet-derived growth factors (PDGF)-BB produced by EPCs enable ADSCs to proliferate and migrate [46, 47]. They also provide proof of physical interaction between ADSCs and ECs in which ECs form a stable tubular, vasculature-like structure with support from ADSCs, both in vitro and in vivo [47]. This information along with some other articles has been used to draw up a schematic in Fig. 1 for the action of SVF, focussing on the interaction between ADSCs and EPCs [46–49].

ADSCs in SVF are currently defined to be positive for classical MSC markers such as CD73 and CD90, and express CD34 but not the pan-haematopoietic lineage marker CD45. CD34 is expressed by progenitors of haematopoietic and endothelial lineages as well, and in ADSCs it is expressed transiently up to about 8–12 population doublings in culture [1].The case of CD34 is interesting since it is still largely considered to be a marker for HSCs owing to its historical association with the enrichment of such cells for bone marrow and umbilical cord blood transplantation. Even the pericytic theory related to MSCs and ADSCs has two sides [50]; whereas Crisen et al. attribute CD34^–^ pericytes to be the progenitors of such stromal cells [51], Traktuev et al. demonstrated a CD34^+^ pericytic identity for ADSCs [46]. Maumus et al. tried to investigate this further but found that native CD34^+^ ADSCs did not exhibit in vivo pericytic markers, but they were rather observed over the course of the culture process [52]. Our data also show that both manually isolated and Stempeutron™-isolated SVF contains a CD146^+^ pericytic population that are mostly (>90%) CD34^–^ [29], suggesting that freshly isolated SVF contains a pericytic population devoid of expressing both CD34 and CD31 markers. Whether the CD146^+^ cells observed within the SVF population subsequently become CD34^+^ ADSCs remains to be determined. Considerable evidence also exists in favour of CD34 expression in bone marrow-derived MSCs (BMMSCs), especially in the early stages of BMMSC research which included data on the disappearance of CD34 upon culturing [53]. Many aspects of this puzzle are yet to be solved, but it is probable that CD34 marks different progenitor cell types such as different MSCs and vascular endothelial progenitor cells.

In the course of preparing this review, it was also observed that reports of ADSC function and physiology in vitro is minimal and in vivo and/or in the native state is rare and in need of further investigation. Table 2 summarises the observations about the characteristics of ADSCs in situ, in vivo, and in vitro that has been discussed within the review [1, 34–36, 46, 47, 52].

**Table 2:** Overview of characteristics of native and culture expanded ADSCs

Factors	Native ADSCs [52]	Culture-expanded ADSCs [1, 34–36, 46, 47, 52]
Cell surface markers	CD34, CD73, CD90, CD13	CD73, CD90, CD13; (CD34 expression decreases and ceases with in vitro expansion)
Morphology	Branched, with up to 80-μm long protrusions forming a network surrounding mature adipocytes	Typical elongated, stromal cell shape, ~10–25 μm and reported to go up to 200 + μm
Location	Both perivascular and stromal positions in situ	Not applicable
Functional characteristics	• Support adipose tissue growth. • Might differentiate to form mature adipocytes	• Facilitate and/or participate in angiogenesis. • Potential for differentiation to adipo-, osteo-, and chondrogenic lineages. • Immunomodulatory properties.

This table summarises important characteristics and differences between native and culture expanded ADSCs [1, 34–36, 46, 47, 52]

*ADSC* adipose-derived stem/stromal cell

### The curious case of CD34

ADSC research, being predominantly carried out using culture-expanded cells, has led to rather recent acceptance of CD34 as a marker for freshly isolated and native ADSCs. Thus, there remain interesting aspects of CD34 biology to be explored and understood. Firstly, CD34 expression has been associated with “stemness” in various systems including human ADSCs. A report by Suga et al. implied association of CD34 expression with naivety, angiogenic gene expression, and greater replicative capacity [54]. Similar to HSCs, reversal of CD34 expression has also been observed in MSCs with a change in culture conditions, thus hinting that CD34 expression might be reversible [53, 54]. Maumus et al. demonstrated an inverse relationship between CD34 expression and in vitro expansion of ADSCs and provided evidence for CD34 being a niche-specific marker of human ADSCs [52]. Interestingly, they commented on the morphological features of ADSCs in vivo, that is having up to 80-μm long protrusions, capable of forming networks surrounding mature adipocytes; however, the scientific and anatomical reason for these structural features are poorly understood. Taking these into account has led to speculation that CD34 is a physiological niche-specific marker of immature/early progenitor cells which is lost in in-vitro conditions [52–56]. Scherberich et al. review CD34 biology in general and with regards to ADSCs in detail [56].

The second interesting aspect is the relationship between CD34 and hypoxia. Since CD34 might be a niche-specific marker of progenitors, it can be speculated that hypoxic conditions might have something to do with its expression. Hypoxia is related to maintenance of adult stem cells such as those in bone marrow and neural stem cells [57]. In MSCs, and also recently in ADSCs, hypoxic pre-conditioning/culturing has shown improved results with regards to proliferation, retention of transplant, angiogenesis, and modulation of angiogenic factors such as VEGF and interleukin (IL)-6, homing, and mobilisation-related characteristics of MSCs/ADSCs, and so forth [58–63]. It is important to note that the ADSC study specifically selected for CD34^–^ cells to begin with and subsequently did not find any significant expression of CD34 in their hypoxically cultured cells [63]. On the other hand, there was a study which speculated that the CD34 gene might be transcriptionally regulated by hypoxia inducible factor 1 (HIF1). The researchers observed that the concentration of oxygen in culture not only influenced the expression of CD34 but also that better maintenance of the antigen corresponded with more undifferentiated cells, which led them to hypothesise that CD34 and hypoxia play an important and inter-related function in maintenance of primitive stem cells of cord blood [64].

Such observations give a certain level of enigma; clearly CD34 and hypoxia are important factors in the maintenance of “stemness”, and it is also likely that CD34 expression is somehow related to hypoxic conditions in different stem or progenitor cell types. However, such a connection remains to be mechanistically studied in human ADSCs, or any other kind of MSCs for that matter. Such studies might provide evidence connecting CD34 with more naive/primitive stem cells, maintained in a hypoxic niche.

## Current state in the clinic and laboratory

The first clinical applications of SVF were reported around 2007 to 2008 for cosmetic breast augmentation and also in the treatment of radiation injury post-radiotherapy in breast cancer patients [14, 65]. The Yoshimura group coined the term CAL, or cell-assisted lipotransfer, in 2008, where they enhanced fat grafts with SVF, demonstrating improved graft retention [14, 17]. Since these two early clinical reports from the last decade, there has been a many-fold increase in basic research and, consequently, many clinical trials are also now underway.

Searching www.ClinicalTrials.gov with keywords such as “SVF”, “Stromal vascular fraction”, “ADSC”, “Adipose stem cells”, and so forth, provides many hits. Although most of those studies are underway or recruiting at the time of this communication, interest has been rising with time. What is truly exciting is the breadth of conditions being targeted by SVF and ADSCs. Despite having properties like MSCs, the use of culture-expanded ADSCs has not reached similar consensus for allogeneic applications. However, ADSCs and SVF have been the preferred regenerative tools for use in autologous applications, and some of the major ones (along with case study references and/or ClinicalTrials.gov identification number) are listed in Table 3 [10, 14, 16, 23, 30, 31, 65–76]. Some other major ailments covered are pulmonary diseases, arterial and vascular diseases, graft versus host disease, Crohn’s disease, peripheral nerve regeneration, and so forth. Clinical areas where SVF and ADSCs are used do overlap to a substantial extent. Nevertheless, there are understandable differences between the two, but the few comparative pre-clinical and clinical studies available do not reach a unanimous conclusion. However, to summarise where the field stands as of now, a comparative overview of both modes with a few examples favouring either option is provided in Table 4 [9, 11, 12, 77].

**Table 3:** Major applications of SVF- and ADSC-based therapeutics with corresponding clinical trials and/or case study references

	Indications	Clinical trials (www.ClinicalTrials.gov)	Case studies and other references
Cosmetic applications	Breast augmentation	NCT02116933	[14, 16, 30, 65, 68, 69]
	General scar, burn and wounds, facial rejuvenation, reconstruction	None found	[10, 23, 65, 70, 71]
	Androgenic alopecia	NCT02594046	None found
Disease conditions	Vulvar lichen sclerosus	None found	[31]
	Erectile dysfunction	NCT02414308, NCT01601353, NCT02087397	[67]
	Peyronie’s disease	NCT02414308	None found
	Urinary incontinence	NCT01799694, NCT01850342	None found
	Faecal incontinence	NCT02292628, NCT01011686	None found
	Anal fistula	None found	[72]
	Multiple Sclerosis	None found	[73]
	Critical limb ischaemia	None found	[74, 75]
	Diabetic foot ulcer	NCT02394886, NCT02092870	None found
	Osteoarthritis	NCT02326961 (Using Celution system)	[76]

This Table provides an overview of major ailments in which ADSCs and SVF are being used therapeutically, with references of case studies and listed clinical trials within www.ClinicalTrials.gov [10, 14, 16, 23, 30, 31, 65–76]

For certain indications, either ClinicalTrial.gov or published cases were not found while preparing this manuscript; this may change in the future

*ADSC* adipose-derived stem/stromal cell, *SVF* stromal vascular fraction

**Table 4:** Comparative overview of SVF and ADSCs

Factors	SVF	ADSCs
Cell population	Heterogeneous	Homogeneous
Cell type	ADSC, EC, EPC, etc.	ADSC only
Application range	Autologous	Autologous & allogeneic
Immune rejection	Not anticipated	Immune monitoring required
Properties	Angiogenic, immunomodulatory, and differentiative	Immunomodulatory and differentiative
Ex vivo exposure	Low (hours)	High (weeks)
Documented advantage in application	Acute myocardial infarction [9] Chronic experimental autoimmune encephalomyelitis [11] Erectile dysfunction in rat model of cavernous nerve injury [12]	Hypertrophic scars [77]

This table provides a comparative overview of ADSCs and SVF with respect to various criteria and lists a few studies which observe advantage of one over the other [9, 11, 12, 77]

*ADSC* adipose-derived stem/stromal cell, *EC* endothelial cell, *EPC* endothelial precursor cell, *SVF* stromal vascular fraction

A superficial glance at the treatments highlights the two most preferred pathways, that is employing the vasculogenic and the immunomodulatory properties. We are yet to fully explore the multipotent properties of SVF cells which will only increase the breadth of their application. One recent example of enhanced osteoinduction by using SVF for dental implant surgery in human subjects provides encouraging results, wherein researchers found bone formation on implanting artificial graft material with SVF supplement compared to the graft alone [66]. The use of matrices/scaffolds and populating those with SVF and/or ADSCs is a promising area of application, though still in experimental phases [13, 78, 79]. Here, we will not go into much detail regarding the applications as that has been well accomplished in a recent two-part review [3, 26].

## “Fat stem cell” therapies and regulatory scenario

Clinics all across the globe began providing “fat stem cell”-based therapy shortly after its discovery, promising miraculous results and more, but often running into controversies [80–86]. Such therapies in the US are known to charge anywhere from USD5000 to USD100,000, and, although mostly harmless and sometimes beneficial, there have been reports of vision loss, tumours, and even deaths [80–86]. Being a major issue in the USA, the FDA had to step in with a draft guideline late in 2014 [87]. These guidelines can be considered in future development of technologies and procedures related to SVF and therapies. Although the “stem cell therapy” genre includes many types of stem cells, ADSCs remain the most marketed variety in the US [88].

The common practices of enzymatic and mechanical disruption of adipose tissue for isolating SVF are explicitly mentioned in the FDA document as “more than minimal manipulation” [87]. As and when the guidelines are implemented, SVF isolated by current protocols (enzymatic digestion) can be treated as a Category 351 product, that is a “drug/biologic” and in need of complete FDA regulation [68]. This calls for exploration of alternate methods, keeping in mind that regulations in the US often trickle down to other geographies, especially in matters of food and drugs.

Introduction of the Reliable and Effective Growth for Regenerative Health Options that Improve Wellness (REGROW) Act [89] in the US Senate last year led to scientific and policy debate, with prominent organisations such as the ISCT, the International Society for Stem Cell Research (ISSCR), and many patient and advocacy groups refusing to support it, at least in its current form [88, 90–94]. The REGROW Act aims to hasten the “conditional approval” of certain cell and tissue therapeutic products which demonstrate “reasonable expectation of effectiveness” along with a few other criteria [89]. However, the use of open-ended terms such as “reasonable expectation of effectiveness” amounts to a lack of clear scientific definition, thus leaving scope for interpretation of the law, consequently leading to potential abuse; such concerns are possibly behind this strong opposition towards the act.

Nevertheless, an urgent consensus is required among all stakeholders with regards to realising the translational potential of stem cells and other cell-based therapeutics, especially when it comes to serious unmet medical needs.

## Conclusions

MSCs have been long known for their remarkable properties when it comes to regeneration and therapeutic potential. ADSCs are possibly the easiest to isolate among all the different types of MSCs in an adult human and in relative abundance too; up to 500 times more stem/stromal cells per gram as compared to a bone marrow source [95]. Simply put, ADSCs are potentially the most abundant regenerative cells in the human body and SVF is a step in the protocol to isolate ADSCs. As has been repeatedly mentioned in this review, the potential for use of both SVF and ADSCs in regenerative medicine are immense. However, care must be taken to go about it without harming the intended beneficiary, that is the patients and public in general. Guidelines, such as the ones from US FDA and their counterparts elsewhere will be important parameters in judging new therapies and technologies being developed, and we ought to keep abreast of such issues. Technology development is the single most important factor to realise the full potential of any new therapy, and SVF-based therapy is no exception. At the same time, it is evident that we need a better understanding of SVF and ADSC biology. This is a continuous endeavour and will only help to better establish the core principles and mechanisms of SVF- and ADSC-based therapies. In the process, we are likely to discover newer applications apart from the plethora already identified. Combining these therapies with other technologies such as decellularised or three-dimensional printed scaffolds with the aim of transplantation will jump-start other areas of clinical and commercial developments.

## Abbreviations

ADSC: Adipose-derived stem/stromal cell
BADSC: Brown adipose-derived stem cell
BAT: Brown adipose tissue
BMMSC: Bone marrow mesenchymal stromal/stem cell
CAL: Cell-assisted lipotransfer
CD: Cluster of differentiation
EC: Endothelial cell
EPC: Endothelial precursor cell
FDA: Food and Drug Administration
HIF1: Hypoxia inducible factor 1
HSC: Haematopoietic stem cell
IFATS: International Federation for Adipose Therapeutics and Science
IL: Interleukin
ISCT: International Society of Cellular Therapy
ISSCR: International Society for Stem Cell Research
MSC: Mesenchymal stem/stromal cell
PDGF: Platelet-derived growth factor
REGROW: Reliable and Effective Growth for Regenerative Health Options that Improve Wellness
SVF: Stromal vascular fraction
VEGF: Vascular endothelial growth factor
VLS: Vulvar lichen sclerosus
WAT: White adipose tissue
